# Atrial fibrillation burden, episode duration and frequency in relation to quality of life in patients with implantable cardiac monitor

**DOI:** 10.1016/j.ijcha.2021.100791

**Published:** 2021-05-11

**Authors:** Victoria Jansson, Lennart Bergfeldt, Jonas Schwieler, Göran Kennebäck, Aigars Rubulis, Steen M. Jensen, Pekka Raatikainen, Elena Sciaraffia, Carina Blomström-Lundqvist

**Affiliations:** a Department of Medical Sciences, Uppsala University, Uppsala SE 751 85, Sweden; b Department of Molecular and Clinical Medicine/Cardiology, Institute of Medicine, Sahlgrenska Academy, University of Gothenburg, and Region Västra Götaland, Department of Cardiology, Sahlgrenska University Hospital, Gothenburg SE 413 45, Sweden; c Department of Cardiology, Karolinska University Hospital, Solna SE 171 76, Stockholm, Sweden; d Heart Centre and Department of Public Health and Clinical Medicine, Umeå University, Umeå SE 901 87, Sweden; e Department of Cardiology, Heart and Lung Center, Helsinki University Hospital, Finland

**Keywords:** Atrial fibrillation, Health-related quality of life, Loop recorder, Implantable cardiac monitor, Atrial fibrillation burden, Randomized

## Abstract

**Aims:**

To assess the relation between atrial fibrillation (AF) characteristics and health-related quality of life (QoL), and which AF characteristic had the greatest impact.

**Method:**

The AF characteristics burden (percentage of time in AF), duration and number of AF episodes/month were obtained from implantable cardiac monitors during the 2-month run-in period in 150 patients included in the randomized CAPTAF trial comparing early ablation and antiarrhythmic drug therapy. The QoL was measured by the General Health and Vitality dimensions of the 36-Item Short-Form Health Survey. AF characteristics were analysed continuously and in quartiles (Q1-Q4).

**Results:**

Greater AF burden (p = 0.003) and longer AF episodes (p = 0.013) were associated with impaired QoL (Vitality score only) in simple linear regression analyses. Greater AF burden was, however, the only AF characteristic associated with lower QoL, when adjusted for sex, type of AF, hypertension, heart rate above 110 beats per minute during AF, and beta-blocker use in multiple linear regression analyses. For every 10% increase in AF burden there was a 1.34-point decrease of Vitality score (95% confidence interval (CI) −2.67 to −0.02, p = 0.047). The Vitality score was 12 points lower (95% CI −22.73 to −1.27, p = 0.03) in patients with an AF burden > 33% (Q4) versus those with < 0.45% (Q1), but only in unadjusted analysis.

**Conclusion:**

AF burden had a greater impact on QoL (Vitality), than the duration and number of AF episodes, corroborating that AF burden may be the preferred outcome measure of rhythm control in trials including relatively healthy AF populations.

## Highlights

• Assessing the relationship between atrial fibrillation and quality of life.
• Implantable loop recorders assessed rhythm continuously in symptomatic patients.
• Atrial fibrillation burden, episode duration and frequency were included.
• Higher atrial fibrillation burden was associated with impaired quality of life.

## 1 Introduction

Atrial fibrillation (AF) is associated with not only increased morbidity and mortality but also with impaired health-related quality of life (QoL) [1], [2]. Furthermore, maintenance of sinus rhythm alleviates symptoms and improves QoL in patients with paroxysmal and persistent AF [3], [4]. Impaired QoL might, however, in the context of AF be due to several factors including sex, severity of symptoms, type of AF, comorbidities and several others [5]. In addition, specific characteristics of AF might have an impact on QoL and this is the topic of the present study.

Prior studies have reported that an AF burden (percentage of time spent in AF) of more than two hours/day, a maximum AF episode duration of more than one hour, and more than 10 episodes/month all predicted impaired QoL [6], [7]. It is not clear, however, if it is the AF burden, the AF episode duration or the AF frequency (number of episodes) that have the greatest impact on symptoms and QoL. In the CAPTAF trial, comparing early ablation versus antiarrhythmic drug therapy, AF burden was found to be inversely related to General Health, a dimension of the 36–Item Short Form Health Survey(SF-36), but other AF characteristics were not analyzed [8]. In the current sub-study of the CAPTAF trial, the aim was therefore to study the association between various AF characteristics and QoL and to identify the AF characteristic with the greatest impact on QoL.

## 2 Method

### 2.1 Study population

The study population comprised 150 of 155 patients with paroxysmal or persistent AF from the CAPTAF trial [8], in which patients were equipped with an implantable cardiac monitor (ICM)(*Reveal®XT: Medtronic Inc., Minneapolis, MN)*. In the present sub-study, the five patients with permanent pacemakers were excluded to have consistent definitions for AF detection. Major inclusion criteria for the CAPTAF trial were age between 30 and 70 years, history of symptomatic AF during the preceding six months, and intolerance to or failure of maximum one antiarrhythmic drug or a beta-blocker. Major exclusion criteria were heart failure (left ventricular ejection fraction < 35%), New York Heart Association functional class (NYHA) III or IV, left atrial diameter > 60 mm, and previous AF ablation.

### 2.2 Design

The design of the CAPTAF trial has been described in detail elsewhere [8] with a protocol available at the European Clinical Trials Database; https://www.clinicaltrialsregister.eu Identifier:2008-001384-11 and at http://ClinicalTrials.gov Identifier:NCT02294955.

In brief, CAPTAF was a prospective multicenter trial including symptomatic AF patients randomized to AF ablation or antiarrhythmic drug therapy. The primary endpoint was General Health, one dimension of the SF-36 [9]. The heart rhythm was continuously monitored by the ICM during a 2-month run-in period prior to randomization and throughout follow-up.

The study was approved by the Ethics Committee and the Swedish Medical Products Agency and conducted according to the Helsinki declaration. Written informed consent was obtained from all patients.

### 2.3 Atrial fibrillation characteristics – burden, duration and frequency

The Reveal® XT automatically detects AF episodes lasting ≥ 2 min by R-wave sensing [10]. In the present study, the ICM data from the 2 months’ run-in period before randomization was used to assess the following AF characteristics (AF burden, episode duration and frequency), and heart rate during AF. The AF burden was defined as percentage of time in AF measured during the run-in period and the AF frequency as the number of episodes per month. The AF burden and frequency were each classified into four quartiles. The median duration of AF episodes in each patient was calculated from the AT/AF Summary report from the ICM and then classified into four groups as close to the quartiles as possible. To adjust for potential inter-individual differences related to differences in rate control, the heart rate during AF was assessed and included in the analyses. The heart rate during AF was dichotomized due to low resolution of data. The classification was based on the median value of the heart rate during AF, according to the Rate histogram report of the ICM in each patient. The cut-off was set to 110 beats per minutes(bpm), which has been defined as an adequate rate control in patients with permanent AF [11].

### 2.4 Health-Related quality of life

Health-Related quality of life (QoL) was assessed by SF-36 at baseline prior to the ICM implantation. The SF-36 consists of eight dimensions reflecting mental and physical components, each with scores ranging from 0 to 100; the lower the score the more impaired QoL. A five point change in the score is considered as “clinically and socially relevant”, 10 points as “moderate” and 20 points as a “very large” difference [9]. A clinically significant difference in QoL score was in the present study defined as more than five points difference. The dimensions General Health(GH) and Vitality(VT) were chosen in the present sub-study, as they were the most affected QoL dimensions when comparing effects of treatment in the CAPTAF trial [(8]. General Health and Vitality measure both a mental and physical component and are two out of four subscales that are regarded as the most precise scales [9]. The reference values for the normal Swedish population for GH and VT are 76.4 and 69.3 [12].

### 2.5 Statistical analyses

Numerical data were expressed as median and interquartile range (IQR Q1-Q3) or as mean ± one standard deviation. Categorical data were expressed as number and proportions (%). Explanatory variables (AF burden, episode duration and frequency) for QoL, expressed both as continuous variables and as quartiles, were evaluated in both simple and multiple linear regression analyses, including adjustment for five variables: sex, heart rate > 110 bpm during AF, hypertension, type of AF(paroxysmal AF and persistent AF), and ongoing beta-blocker medication.

All analyses were performed using R statistics software (Version 3.5.2;R Foundation for Statistical Computing, Vienna, Austria) and package Rcmdr (Version 2.6–0). P-values < 0.05 were considered statistically significant. P-values were not adjusted for multiple testing as the study was exploratory.

## 3 Results

### 3.1 Demographic data and clinical characteristics

The clinical characteristics of the cohort are presented in Table 1. The majority was men, the mean age was 58 years, most patients had paroxysmal AF, and hypertension was the most common concomitant disease. The run-in period and time used for rhythm monitoring by the ICM was 2.03 ± 0.46 months. Fourteen patients (9%) had no detected AF episode during the recorded period and 28 patients (19%) had an AF burden ≤ 1%. Thirteen patients had an AF burden of 100%. In about one third of the study population (52 patients) the median AF episode duration was 6 min. The number of AF episodes varied from none to 719 per month (median 3.6, IQR 0.98–16.40).

**Table 1:** Patient characteristics.

Demographic variables	Patients, n = 150 (%)
Age (years), median	58.0 (50.0–64.0)
Sex, male	116 (77)
BMI (kg/m^2^), median	26.6 (24.6–29.0)
Paroxysmal AF	108 (72)
Persistent AF	41 (28)
AF history diagnosed by history (years), median	3.5 (1.5–7.4)
Median heart rate during AF > 110 bpm*	27 (22)
Ongoing beta-blocker medication	92 (61)
Ongoing antiarrhythmic drug	40 (27)
Number of AF episodes during last 12 months, median	7.5 (4.0–48.0)
Left atrial diameter (cm)/BSA, median	2.01 (1.82–2.17)
Left ventricular ejection fraction (%), mean	56.2 ± 7.4
CHA_2_DS_2_-VASc score, mean	1.02 ± 1.10
Comorbidities	
Hypertension	59 (39)
Bradycardia or SSS	10 (7)
Diabetes mellitus	6 (4)
Chronic lung disease	8 (5)
Coronary artery disease	5 (3)
Heart failure	5 (3)
Sleep apnoea	5 (3)
Stroke/TIA/peripheral emboli	4 (3)
Valvular disease	2 (1)

Data are expressed as number of patients (%) unless otherwise stated as median (interquartile range) or mean ± standard deviation. * = missing data of 25 patients.

AF = Atrial fibrillation; BSA = Body surface area, calculated with DuBois & DuBois formula; SSS = Sick Sinus Syndrome; TIA = Transient ischemic attack.

### 3.2 Atrial fibrillation characteristics and quality of life scores

The median AF burden was 3.6% (IQR 0.45–32.90%), the median AF episode duration 35 min (IQR 6-1080 min), and the median AF frequency 3.6 episodes per month (IQR 0.97–16.50). The median GH score was 65 points (IQR 47–77) and the median VT score was 60 points (IQR 35–75), both of which are on average 10 points below the Swedish reference population, thus confirming impaired QoL.

**Fig. 1:**
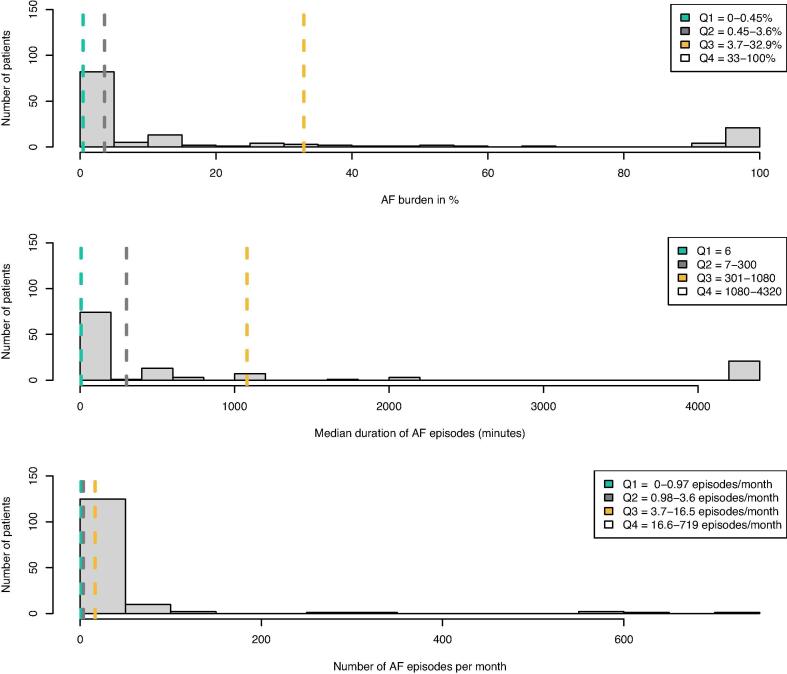
Histogram of AF burden, episode duration and frequency with quartiles (Q1-Q4) outlined. The figure demonstrates a histogram of the AF characteristics for the whole study population of 150 patients, with the quartiles outlined. In 52 patients the median duration of AF episodes was 6 min (Q1) and hence Q2 included 23 patients, Q3 included 23 patients and Q4 included 25 patients. In both AF burden and frequency, Q1, Q2 and Q3 included 36 patients while Q4 included 35 patients. AF = Atrial fibrillation; AF burden = % of time spent in AF;

The patients’ AF burden, episode duration and frequency were classified into quartiles (Fig. 1). Within the fourth AF burden quartile 25 out of 35 patients had persistent AF. Within the first AF frequency quartile 17 out of 36 patients had persistent AF and within the fourth AF frequency quartile 4 out of 35 patients had persistent AF.

### 3.3 Unadjusted models

In the simple linear regression analyses including analysis of quartiles, neither AF burden, AF episode duration nor AF frequency were associated with GH score (Table 2 and Table 3). An inverse relation was, however, found between AF burden and VT score. For each 10% increase in AF burden, the VT score decreased by 1.58 points (−2.60 to −0.55, p = 0.003) (Table 2). Hence, a clinically significant improvement in VT score, defined as five points, would correspond to about 32% decrease in AF burden. The fourth quartile of AF burden (33–100% of time in AF) was associated with a 12 points lower VT score (95% CI −22.73 to −1.27, p = 0.03) than the first quartile (0–0.45% of time in AF) (Table 3).

**Table 2:** Atrial fibrillation burden, episode duration and frequency as continuous variables associated with health-related quality of life in adjusted and unadjusted models.

	Simple regression analyses (141 patients)				Multiple regression analyses^a^ (114 patients)			
	General health		Vitality		General health		Vitality	
	Estimate (95% CI)	P-value	Estimate (95% CI)	P-value	Estimate (95% CI)	P-value	Estimate (95% CI)	P-value
AF burden, %^b^	−0.65 (−1.54–0.26)	0.16	−1.58 (−2.60 to −0.55)	0.003	−1.00 (−2.19–0.18)	0.10	−1.34 (−2.67 to −0.02)	0.047
AF episode duration, minutes^c^	−0.04 (−0.17–0.09)	0.57	−0.20 (−0.35 to −0.04)	0.013	−0.03 (−0.20–0.14)	0.73	−0.13 (−0.32–0.07)	0.21
AF frequency, episodes per month	0.01 (−0.02–0.04)	0.34	0.02 (−0.02–0.05)	0.35	0.02 (−0.02–0.05)	0.45	0.01 (−0.03–0.05)	0.63

AF = Atrial fibrillation; AF burden = % of time spent in AF; AF episode duration = AF episode duration in minutes; AF frequency = AF episodes/month; CI = Confidence interval; General health and Vitality = dimensions in the 36–Item Short Form Health Survey (SF-36).

a Models adjusted for sex, heart rate > 110 beats per minute during AF, hypertension, type of AF and ongoing beta-blocker medication.

b With 10% increase.

c With 1-hour increase and 122 patients included in simple regression analyses and 106 patients in multiple regression.

**Table 3:** Atrial fibrillation burden, episode duration and frequency as variables associated with quality of life, when divided in quartiles, unadjusted models.

	Simple regression analyses (141 patients)					
	General health			Vitality		
Quartiles	Estimate (95% CI)	P-value	Group P-value	Estimate (95% CI)	P-value	Group P-value
*AF burden*						
Q1: 0–0.45%	Reference					
Q2: 0.45–3.6%	−0.16 (−9.44–9.12)	0.972	0.55	3.14 (−7.44–13.72)	0.558	0.03
Q3: 3.7–32.9%	−4.41 (−13.69–4.87)	0.349		0.22 (−10.35–10.8)	0.967	
Q4: 33–100%	−5.42 (−14.83–4)	0.257		−12.00 (−22.73 to −1.27)	0.029	
*AF episode duration*^b^						
Q1: <6 min	Reference					
Q2: 7–300 min	2.76 (−6.49–12.01)	0.555	0.14	−3.05 (−14.36–8.26)	0.595	0.17
Q3: 301–1080 min	−9.28 (−18.53 to −0.04)	0.049		−5.22 (−16.53–6.09)	0.363	
Q4: 1080–4320 min	−2.30 (−11.41–6.81)	0.618		−12.68 (−23.82 to −1.53)	0.026	
*AF frequency*						
Q1: 0–0.97 episodes/mo	Reference					
Q2: 0.98–3.6 episodes/mo	−11.80 (−20.9 to −2.7)	0.011	0.06	−4.30 (−15.14–6.54)	0.434	0.55
Q3: 3.7–16.5 episodes/mo	−2.83 (−12–6.34)	0.543		3.29 (−7.63–14.2)	0.553	
Q4: 16.6–719 episodes/mo	−2.6 (−11.77–6.57)	0.576		1.57 (−9.35–12.49)	0.776	

Abbreviations **as in**Table 2. Min = minutes; mo = month.

b = with 1-hour increase and 122 patients included in simple regression analyses.

An inverse relationship was found between AF episode duration and VT score; for every 1-hour increase in median duration the VT score decreased 0.20 points (95% CI −0.35 to −0.04, p = 0.013) (Table 2). When comparing AF episode duration and AF frequency quartiles on one hand with the VT score on the other, there were no statistically significant relation.

### 3.4 Multiple linear regression analyses

After adjusting for sex, heart rate > 110 bpm during AF, hypertension, type of AF and ongoing beta-blocker medication, only AF burden was inversely related to the VT score (Table 2). For every 10% increase in AF burden the VT score decreased by 1.34 points (95% CI 2.67 to −0.02, p = 0.047) (Table 2). When the adjusting variables were included in the analysis based on quartiles, none of the AF characteristics were significantly associated with the QoL scores.

## 4 Discussion

### 4.1 Atrial fibrillation characteristics

The impact of AF burden, episode duration and frequency on QoL was investigated in this sub-study of the randomized CAPTAF trial. No prior study has to our best knowledge evaluated which of these three AF characteristics has the greatest impact on the QoL measures GH and VT of the SF-36. The main finding was that AF burden had the greatest impact on VT score. Only AF burden remained independently and inversely associated with VT score after adjusting for several cofounding factors.

In the present study, an AF burden > 33% (eight hours daily) was associated with a reduced QoL, which is higher than > 2 h reported to affect the mental and physical score using the shorter SF-12 in a previous study [6]. There are several potential explanations for these differences. First, our study population was younger with less comorbidities, which may explain the higher AF burden required to affect QoL. Our cohort had a normal left ventricular ejection fraction and low prevalence(3%) of heart failure, in contrast to the other study [6], reporting 20% prevalence. Moreover, while the current study adjusted for heart rate during AF, sex, type of AF, and beta-blocker medication, that study [6] adjusted for age, sex, and history of heart failure but not for type of AF, heart rate during AF, and beta-blocker medication. There was also a methodological difference [6], as dual chamber pacemakers were used, whereas in the current study the analyses were based on recordings from ICMs, which may have contributed to the divergent results. Finally, the different results may also be related to their use of a shorter QoL questionnaire [6].

In another study, a monthly AF burden of > 4.8 h at follow-up after AF ablation had an impact on all dimensions of SF-36 [13]. AF burden was estimated from clinical history, external loop recorders and intermittent 7 day Holter monitoring, which may have underestimated the degree of AF burden and, thus, makes a comparison to the current study difficult [14].

In patients referred for catheter ablation [7], a longest AF episode duration of > 1 h predicted a change in the mental components of QoL and > 10 episodes/month predicted a change in the physical components using SF-36. In the present study, AF episode duration was associated with lower VT score in simple linear regression analysis but not when adjusted. Dichotomous variables of AF characteristics were used to predict the effects on QoL [7], while the current study used continuous variables when feasible in addition to analysis based on quartile groups. The differences in method and measured QoL dimensions may explain the divergent results.

The association between increasing AF burden and decreasing QoL score could only be demonstrated for the subscale VT but not for GH, which might seem to contradict the results from the CAPTAF trial showing a relation between improved GH and decreased AF burden [8]. In the CAPTAF trial, however, the finding of greater improvement in QoL relating to the greater reduction in AF burden was seen after treatment intervention versus baseline. The differences in GH or AF burden during run-in in the whole cohort may have been too small or too widespread to disclose such an inverse relationship. Moreover, the odd or wide distribution of AF burden in the present cohort, which may be explained by the short recording period and the mixed types of AF, may be another reason for not being able to disclose a relationship between GH and AF burden.

### 4.2 Impact on quality of life

Quality of Life is affected by many variables. To prove a causal connection between an AF episode, being asymptomatic or symptomatic, and its direct effects on QoL is challenging. Younger patients report more prominent impairment of QoL than older [15], female patients report more pronounced symptoms than male [16], and patients with paroxysmal AF report more symptoms than those with persistent or permanent AF [17]. Our observation that increasing AF burden is associated with worse QoL in patients with non-permanent AF, does not contradict previous findings that permanent AF patients (100% AF burden) are less symptomatic than those with paroxysmal or persistent AF for 2 reasons. First, our findings refer to AF patients who experience periods of sinus rhythm, and thus without increasing tolerance or adaption to an ongoing chronic arrhythmia, as is the case for permanent AF [18]. Second, since patients with permanent AF in general are older, have more comorbidities, and are less active, their requirements on physical capacity are lower.

### 4.3 Clinical implications and future applications

Symptom related impairment in QoL is the major indication for catheter ablation in AF patients [19]. The present study suggests that AF burden has the greatest impact on QoL, as compared with other measures of AF including episode duration and frequency. We believe that it was an advantage to study a population with few comorbidities as in the present cohort, since it limited confounding factors that may affect QoL assessments, apart from AF per se. The present findings have potential implications for future recommendations on which outcome measure of rhythm should be recommended in clinical practice and future trials. The AF guidelines currently define recurrence after AF ablation as a 30-seconds AF episode, although questions have been raised whether such arbitrary defined variable should be abandoned and replaced by AF burden when assessing outcomes in AF ablation [19].

## 5 Limitations

The ICMs were implanted after the completion of the SF-36 in order not to influence the outcome of the questionnaires in the main study. This is a potential limitation in this sub-study since the patient was asked to reflect on the QoL during the last 4 weeks [9]. The AF pattern was, however, expected to be fairly similar before QoL assessment and during ICM recording, since there was no change in treatment. The results may not be applicable to AF-populations in later stages of the AF disease, since the present study mainly included a relatively young, healthy population with an early stage of the AF disease.

## 6 Conclusion

Atrial fibrillation burden had the greatest impact on QoL represented by the Vitality sub-scale in 36–Item Short Form Health Survey (SF-36), as opposed to AF episode duration and frequency in symptomatic AF patients. AF burden might therefore be a more appropriate outcome measure of rhythm control than the currently used 30-second AF episode in future trials including relatively healthy AF populations.

## Declaration of Competing Interest

Dr Blomström-Lundqvist reports receiving grants from Medtronic during the conduct of the study; and personal fees from Bayer, Sanofi, Boston Scientific, and Merck Sharp & Dohme outside the submitted work. Dr Bergfeldt reports receiving personal fees from Sanofi, Bristol-Myers Squibb, Bayer, and Pfizer outside the submitted work. Dr Raatikainen reports receiving grants from Biosense Webster outside the submitted work. All remaining authors have declared no conflicts of interest.
